# The association of hepatitis c virus infection status with serum glucose levels

**DOI:** 10.1186/s12876-019-1003-3

**Published:** 2019-06-13

**Authors:** Yinping Li, Xiaomei Wang, Ge Yu, Haibo Sun, Juan Lv, Xiumei Chi, Ruihong Wu, Xiuzhu Gao, Junqi Niu

**Affiliations:** 1 Department of Hepatology, the First hospital of Jilin University, No.71 Xinmin Str, Changchun, 130021 China; 2 Department of Gastroenterology, Shouguang City People’s Hospital, Shouguang, 262700 China; 3 Key Laboratory of Zoonosis Research, Ministry Education, Changchun, 130021 China

**Keywords:** Chronic hepatitis C, Antiviral treatment, Fasting blood glucose, Sustained virological response

## Abstract

**Background:**

Hepatitis C virus (HCV) infection is commonly associated with a disturbance of glucose metabolism. However, there have been conflicting reports on whether the clearance of the HCV may be followed by changes of serum blood glucose and insulin resistance. The aim of the present study was to evaluate the impact of HCV and antiviral treatment on serum glucose levels and other glucose metabolism parameters.

**Methods:**

A retrospective analysis of 306 HCV-infected patients was performed. Fasting serum blood glucose (FBG) levels in these patients were compared with that of 325 healthy individuals. Serum parameters of glucose metabolism were measured in 183 patients with chronic hepatitis C at baseline, at the end of interferon α-2b plus ribavirin treatment, and at 24 weeks post-treatment.

**Results:**

Patients with HCV infection had significantly higher FBG level than healthy controls (5.57 ± 0.74 vs. 5.11 ± 0.83 mmol/l, *P* < 0.001). After antiviral treatment, we found a significant reduction in FBG levels regardless of the outcome of treatment. However, after stopping treatment the serum FBG levels were significantly elevated in the sustained virological response (SVR) and non-responder groups, and maintained high level until week 24 post-treatment. In both groups, the levels of serum FBG after 24 weeks post-treatment were still lower than pre-treatment levels. In sustained responders, fasting insulin (*P* = 0.007), C-peptide (*P* < 0.001) and HOMA-IR (*P* < 0.001) significantly decreased, and the insulin sensitivity index (ISI) increased (*P* < 0.001) at the end of the treatment comparing with pre-treatment levels, while no significant difference was observed in non-responder group. HOMA-β values were increased in both groups at the end of treatment (both *P* < 0.001).

**Conclusion:**

The total serum FBG level in HCV infected patients was higher than that in healthy controls. Clearance of HCV was associated with reduced glucose and improved insulin resistance.

## Background

Hepatitis C virus (HCV) infects over 170 million individuals worldwide, and is an important cause of liver cirrhosis and hepatocellular carcinoma [1]. Currently, the therapy agents mainly consist of direct-acting agents such as protease, polymerase, and polymerase accessory protein inhibitors, while the interferon (IFN) and ribavirin combination therapy still plays an important role, especially in some developing countries [2].

Although HCV is a hepatotropic virus, it has also been identified in extrahepatic tissues. Numerous extrahepatic manifestations have been reported to be related to HCV infection, including mixed cryoglobulinemia, thyroiditis, a high prevalence of autoantibodies and metabolic disorders [3, 4]. Recently, much attention focuses on the association between HCV infection and the glucose intolerance [5–7]. It was believed that HCV infection could induce insulin resistance (IR). Some studies showed that the prevalence of IR ranged from 30 to 70% in HCV-infected patients regardless of the severities of liver disease, while HCV eradication could induce a decreased risk of insulin resistance [8–11]. However, other studies reported that there was no association between HCV clearance and metabolic syndrome [12].

In this study, we aimed to investigate the association of HCV infection with serum glucose levels, and the impact of antiviral treatment on glucose metabolism.

## Methods

### Subjects

A total of 631 Chinese Han subjects were enrolled in this study, including 306 chronic hepatitis C (CHC) patients and 325 healthy controls. All subjects were originally included in a previous epidemiological investigation of HCV infection carried out by the First Hospital of Jilin University in Fuyu county of Jilin Province from 2012 to 2014. Chronic HCV infection was defined as anti-HCV positive and detectable serum HCV RNA for more than 6 months. Patients with concomitant human immunodeficiency virus, co-infection of hepatitis B virus, liver cirrhosis or diabetes mellitus were excluded. Patients who were currently receiving or recently received anti-viral treatment were also excluded. The healthy controls satisfied the following criterion: (a) body mass index (BMI) < 30 kg/m^2^; (b) anti-HCV was negative; (c) fasting glucose < 6.10 mmol/l. The healthy controls were also matched to CHC by age, sex and body mass index (BMI). Subjects with any liver disease or diabetes history were excluded. Baseline data including age, gender, body mass index (BMI), serum fasting blooding glucose (FBG), aspartate transaminase (AST), alanine transaminase (ALT), alkaline phosphatase (ALP) and gamma-glutamyltransferase (GGT) were collected. BMI was calculated as weight in kilograms divided by height in square meters.

From the 306 CHC patients, 183 patients were selected for treatment after screening for medical and mental health-related contraindications, and were offered recombinant interferon α-2b (500,000 IU, 3 times/week, Beijing Kavin Technology Share-holding Co, Ltd. China) subcutaneously, and oral ribavirin (15 mg/kg/day) over 48 weeks (for genotype 1 and 2). This antiviral combination was selected to be studied for economic reasons. Patients were monitored until 24 weeks after the end of the treatment. All of the 183 treated patients were compliant with medications taking at least 80% of the dose and in 80% of the time, and the results of this group were analyzed.

The 183 treated patients were divided into two groups according to their virological response. Sustained virological response (SVR) was defined as an undetectable HCV RNA during at least six months after completion of therapy.

The study protocol was approved by the ethics committee of the First Hospital of Jilin University. Written informed consent for all testing was obtained prior to enrollment.

### Laboratory investigations

Blood samples were obtained in the morning after more than 8 h fasting, and measured in the Clinical Laboratory of the First Hospital of Jilin University. The tests for biochemistry, liver function, renal function, blood glucose, fasting insulin and C peptide were performed using a Synchron LX®20 autoanalyser (Beckman Coulter, Brea, CA, USA). HCV RNA was determined by quantitative real-time polymerase chain reaction using the COBAS AmpliPrep/COBAS TaqMan (Roche Diagnostics, Mannheim, Germany), and the lower detection limit was 15 IU/ml. Level of anti-HCV was measured using an Abbott ARCHITECT i2000SR. HCV genotype performed using multicolor fluorescence PCR with an HCV RNA genotype kit (BioAssay Science & Technology Co. Ltd., China).

HCV viral load, HCV genotype, serum FBG, fasting insulin levels (FINS) and fasting C peptide (FCP) were collected respectively at baseline, at the end of treatment and at week 24 post-treatment. Three parameters of glucose metabolism, including homeostasis model assessment of insulin resistance (HOMA-IR), homeostasis model assessment- β (HOMA-β) and insulin sensitivity index (ISI), were evaluated by the homeostasis model assessment and were calculated as follows:

HOMA-IR = FINS (μU/ml) × FBG (mmol/l)/22.5.

HOMA-β = 20 × FINS (μU/ml)/(FBG (mmol/l)-3.5).

ISI = ln 1/FINS (μU/ml) × FBG (mmol/l).

### Statistical analysis

Continuous variables were presented as means ± SD or median (inter-quartile range), while categorical variables were expressed as frequencies (%). Continuous variables between groups were analyzed using a Student *t*-test or Mann–Whitney U test while categorical variables were analyzed using a chi-squared test. HCV RNA levels were expressed as the mean ± standard deviation after logarithmic transformation of original values. Multivariate logistic regression was performed as appropriate. The procedures were performed using the SPSS 18.0 statistical package. All statistical analyses were based on two side hypothesis tests, with values *P* < 0.05 considered to indicate statistical significance.

## Results

### Demographic and clinical characteristics of the HCV-infected patients and the healthy control group

Demographic and clinical characteristics of the HCV-infected patients and the healthy control groups were presented in Table 1. There was no significant difference in age, gender and BMI between two groups (*P* = 0.143; *P* = 0.129 and *P* = 0.247, respectively). HCV-infected group had a significantly higher baseline fasting blood glucose levels than healthy control (5.57 ± 0.74 vs. 5.11 ± 0.83, *P* < 0.001). AST, ALT, ALP and GGT levels were also significantly higher in HCV group (*P* < 0.001 for all).

**Table 1:** Baseline characteristics of chronic hepatitis C patients and controls

Characteristic	HCV	Control	*P value*
N	306	325	
Male (%)	191 (62.4)	179 (55.1)	0.129
Mean age (years)	46.34 ± 5.42	45.97 ± 6.22	0.143
BMI	23.27 ± 3.14	23.53 ± 2.59	0.247
FBG (mmol/l)	5.57 ± 0.74	5.11 ± 0.83	< 0.001
ALT (U/L)	50.4 (29.8–80.35)	27.4 (18.7–42.65)	< 0.001
AST (U/L)	38.0 (27.65–62.8)	24.85 (19.8–33.8)	< 0.001
ALP (U/L)	79.0 (64.0–95.0)	77.0 (63.0–93.0)	< 0.001
GGT (U/L)	40.0 (23.0–96.0)	24.5 (16.0–47.45)	< 0.001

Data are expressed as mean ± SD, median (interquartile range) or as number of patients

*BMI* body mass index, *FBG* fasting blood glucose, *ALT* alanine transaminase, *AST* aspartate transaminase, *ALP* alkaline phosphatase, *GGT* gamma-glutamy ltranspeptidase

### Baseline characteristics of SVR and non-SVR patients

Baseline characteristics of 183 HCV-infected patients are presented in Table 2. SVR was obtained in 59.6% (*n* = 109), and non-SVR in 40.4% (*n* = 74) of the 183 patients. There was no significant difference in gender and BMI between the two groups. Most HCV genotypes of the patients were 1b or 2a, except 3 patients whose HCV genotypes were not determined. Significant difference was observed in age, genotype, HCV load, ALT and AST levels between SVR and non-SVR groups. The predictors of SVR were young age (*P* = 0.008), non-genotype 1 (*P* < 0.001), low HCV load (*P* < 0.001), high ALT (*P* = 0.038) and AST levels (*P* = 0.033) with univariate analysis. However, baseline FBG, INS and HOMA-IR negatively affected treatment response (*P* = 0.159, 0.534, 0.314).

**Table 2:** Baseline Characteristics of the Treated HCV Group

	SVR	non-SVR	*P value*
N	109	74	
Male(%)	79 (72.5%)	51 (68.9%)	0.622
Mean age (years)	48.4 ± 7.82	51.8 ± 7.41	0.008
BMI	23.7 ± 2.72	23.7 ± 3.02	0.798
HCV load (Log_10_)	5.64 ± 1.01	6.35 ± 0.76	< 0.001
HCV genotype (%)			
1b	50 (45.9%)	61 (82.4%)	< 0.001
2a	56 (51.4%)	13 (17.6%)	
Unclassified	3 (2.7%)	–	
FBG (mmol/l)	5.14 ± 0.81	5.52 ± 0.83	0.159
FCP (nmol/l)	0.92 ± 0.25	0.95 ± 0.33	0.409
FINS (μU/ml)	8.30 ± 3.80	9.08 ± 4.88	0.534
HOMA-IR	2.00 ± 1.02	2.27 ± 1.37	0.314
HOMA-β	104.5 ± 71.92	104.5 ± 77.53	0.9
ISI	−3.70 ± 0.45	−3.77 ± 0.56	0.353
ALT (U/L)	64.0 (34.3, 131.5)	49.6 (30.7, 72.6)	0.038
AST (U/L)	49.4 (32.2, 75.5)	39.9 (30.5, 62.6)	0.033
ALP (U/L)	85.0 (70.5, 113.5)	86.0 (64.8, 106.5)	0.642
GGT (U/L)	45.0 (25.3, 90.8)	44.0 (23.3, 86.3)	0.731
ALB (g/l)	48.2 (44.7, 52.5)	46.9 (43.9, 50.0)	0.064
TBIL (μmol/l)	17.8 (12.9, 28.2)	15.9 (11.3, 23.0)	0.529
CHE (U/L)	7598 (6721, 8030)	7835 (6870, 8148)	0.09

Data are expressed as mean ± SD, median (interquartile range) or as number of patients

*BMI* body mass index, *TC* total cholesterol, *TG* triglyceride, *FBG* fasting blood glucose, *FCP* fasting C peptide, *FINS* fasting insulin, *HOMA-IR* homeostasis model assessment for insulin resistance, *HOMA-β* Homeostasis model assessment for beta-cell function, *ISI* insulin sensitivity index, *ALT* alanine transaminase, *AST* aspartate transaminase, *ALP* alkaline phosphatase, *GGT* gamma-glutamyltransferase, *ALB* albumin, *TBIL* total bilirubin, *CHE* cholinesterase

Multiple regression analysis found that old age (*P* = 0.004, OR = 1.072,95% CI: 1.023, 1.123), high viral load (*P* < 0.001, OR = 2.316, 95% CI: 1.596, 3.362) and genotype 1b (*P* < 0.001, OR = 2.016, 95% CI: 1.116, 3.122) were predictive of a poor virological response.

### Changes in insulin resistance and beta-cell function after antiviral therapy in SVR and non-SVR group

To investigate the effects of HCV clearance on glucose metabolism, we compared some of the metabolic indices of SVR and non-SVR group. Changes in insulin resistance and beta-cell function after antiviral therapy in SVR and non-SVR patients are summarized in Tables 3 and 4. In SVR group, serum FBG, FINS and FCP levels significantly decreased after the end of treatment (from 5.41 ± 0.8 to 4.60 ± 0.68 mmol/l, *P* < 0.001; from 8.30 ± 3.80 to 7.88 ± 4.15 μU/ml, *P* = 0.007; from 0.92 ± 0.25 to 0.72 ± 0.26 nmol/l, *P* < 0.001, respectively). HOMA-IR values also decreased at the end of treatment (from 2.00 ± 1.02 to 1.62 ± 0.94, *P* < 0.001), and rebounded to 1.86 ± 1.20 at 24 weeks after treatment. Correspondingly, ISI values increased from − 3.70 ± 0.45 at baseline to − 3.45 ± 0.54 (Table 3). However, no significant changes in both parameters of glucose metabolism and HOMA-IR values were observed in non-SVR group (Table 4).

**Table 3:** Time course of changes in serum beta-cell function during antiviral treatment of patients with SVR

	Baseline	End-Rx	FU-24	*P*^*1*^ value	*P*^*2*^ value
FBG (mmol/l)	5.41 ± 0.81	4.60 ± 0.68	5.09 ± 0.83	< 0.001	0.002
FINS (μU /ml)	8.30 ± 3.80	7.88 ± 4.15	8.18 ± 3.99	0.007	0.806
FCP (nmol/l)	0.92 ± 0.25	0.72 ± 0.26	0.78 ± 0.27	< 0.001	0.000
HOMA-IR	2.00 ± 1.02	1.62 ± 0.94	1.86 ± 1.20	< 0.001	0.052
HOMA-β	104.5 ± 71.9	183.7 ± 127.8	134.3 ± 108.6	< 0.001	0.01
ISI	−3.70 ± 0.45	−3.45 ± 0.54	−3.60 ± 0.52	< 0.001	0.05

Data are expressed as mean ± SD;

*FBG* fasting blood glucose, *FINS* fasting insulin, *FCP* fasting C peptide, *HOMA-IR* homeostasis model assessment for insulin resistance, *HOMA-β* homeostasis model assessment for beta-cell function, *ISI* insulin sensitivity index, *Rx* treatment; FU-24, follow up at 24 weeks post treatment

*P*^*1*^*,* value for comparison of baseline and end-Rx values; *P*^*2*^*,* value for comparison of baseline and FU-24 values

**Table 4:** Time course of changes in serum beta-cell function during antiviral treatment of patients non- SVR

	Baseline	End-Rx	FU-24	*P*^*1*^ value	*P*^*2*^ value
FBG (mmol/l)	5.52 ± 0.83	4.93 ± 0.93	5.23 ± 0.74	< 0.001	0.023
FINS (μU/ml)	9.08 ± 4.88	9.05 ± 5.07	9.58 ± 5.12	0.546	0.888
FCP (nmol/l)	0.95 ± 0.33	0.97 ± 0.29	0.93 ± 0.31	0.609	0.819
HOMA-IR	2.27 ± 1.37	2.16 ± 1.42	2.49 ± 1.37	0.281	0.195
HOMA-β	104.5 ± 77.5	178.7 ± 148.3	144.3 ± 102.9	< 0.001	0.08
ISI	−3.77 ± 0.56	−3.69 ± 0.63	−3.87 ± 0.59	0.250	0.264

Data are expressed as mean ± SD;

*FBG* fasting blood glucose, *FINS* fasting insulin, *FCP* fasting C peptide, *HOMA-IR* homeostasis model assessment for insulin resistance, *HOMA-β* homeostasis model assessment for beta-cell function, *ISI* insulin sensitivity index, *Rx* treatment; FU-24, follow up at week 24 post treatment

*P*^*1*^*,* value for comparison of baseline and end-Rx values; *P*^*2*^*,* value for comparison of baseline and FU-24 values

HOMA-β values in both groups were obviously increased at the end of treatment compared with baseline (*P* < 0.001 for both). The values decreased slightly at 24 week after end of treatment, but were still higher than pre-treatment levels (*P* = 0.01 and *P* = 0.08, respectively).

## Discussion

In the present study, we evaluated the association of HCV infection and glucose metabolism; the effect of antiviral treatment on glucose metabolism was also studied [13].

Our results showed that the level of FBG in HCV group was higher than that in control group, which was consistent with other studies. Previous studies has revealed that HCV infection causes insulin resistance and glucose abnormalities even type 2 diabetes mellitus (T2DM) in susceptible individuals [11, 14]. Another long period follow-up study showed the cumulative incidence of T2DM in anti-HCV positive patients reached 14.3% while only 8.6% in seronegative individuals (*P* < 0.0001) [15].

The relation between IR and SVR are controversy. Our study revealed that many factors at baseline such as age, HCV genotype, HCV load, ALT and AST levels were able to predict SVR, but there was no significant association between FBG, INS, HOMA-IR with SVR. A meta-analysis for the association between insulin resistance and SVR in hepatitis-C infected patients indicated that there was no relation between them and the mean value of HOMA-IR was less than 3 at baseline in all studies [16]. Other studies reported that pretreatment glucose intolerance and IR could impair the therapy outcome [17, 18]. The association of IR and SVR needs further prospective studies.

Clearance of HCV infection may improve IR and increase insulin sensitivity. In our study, we found that in SVR patients eradication of HCV by interferon-based therapy could improve insulin resistant and decreased fasting insulin as well as C peptide levels, whereas no significant difference in these indices was found in non-SVR patient. This result suggested that HCV itself might be involved in the development of insulin resistance. The mechanisms that HCV induces glucose metabolism abnormities are not very clear.

Several different mechanisms may be involved. HCV-induced liver inflammation and cirrhosis may reduce the uptake of glucose by hepatic cells, then affect the glucose metabolism [19]; HCV infection may also impair IRS-1 tyrosine phosphorylation. As anessential molecule in insulin signaling, the dysfunction of IRS-1 could decrease downstream insulin effects, thereby contributing to glucose intolerance. Clearance of HCV results in a significant increase of IRS-1 expression, which may partly restore glucose metabolism [7, 20]. In addition, HCV core proteins may functionally inhibit insulin signaling via increasing the level of tumor necrosis factor-alpha (TNF-α), which is an important factor mediating insulin signaling [21]. HCV core proteins also down regulate the cell surface expression of glucose transporter 2 (GLUT2), promoting hepatic gluconeogenesis via suppression of glucose uptake [19, 22]. Finally, some proinflammatory cytokines such as IL-6 and TGF-β also have been identified to be able to reduce glucose uptake and impair glucagon metabolism in HCV infected patients.

Our study also showed that HOMA-β was significantly increased after antiviral treatment regardless of the outcome. The mechanism may be attribute to the restoration of insulin sensitivity after the clearance of HCV [23]. The current data confirmed that HOMA-β values increased in the CHC patients as compensation for insulin resistance [24, 25], and at the same time the status of beta-cell hyperfunction was ameliorated after successful treatment [26]. The mechanisms that whether or not HCV could change the function of beta-cell in chronic hepatitis C patients remains unclear. Early studies reported that virus-like particles were detected in the pancreatic beta cells of HCV patients, which could induce death of pancreatic beta cells through multiple mechanisms [27, 28]. While many clinical studies reported that beta-cell function was preserved [29].

Larger amount of samples was included in our study than other studies [2, 14], which increased the statistical reliability of the results. Although the therapy agents in the study were no longer generally used for the treatment of HCV, the interferon and ribavirin combination therapy still plays an important role in certain limited resource settings. There were also certain limitations. The current study was retrospective, and some data missed in some cases. However, we proposed that no matter what kind of antiviral treatment was used, clearance of HCV could improve insulin resistant and decrease fasting glucose [11, 20]. It is still interesting to study the impact of conventional methods on metabolic endpoints.

## Conclusions

The current study showed that chronic HCV-infected patients had higher fasting blood glucose levels than healthy controls. Successful clearance of HCV by interferon-based therapy led to reversal of hyperglycemia and improvement of insulin resistance However, the effect of HCV infection on beta cell function is complex and further studies are required to clarify their association.

## Data Availability

The datasets used and analyzed during the current study are available from the corresponding author on reasonable request.

## Abbreviations

ALP: Alkaline phosphatase
ALT: Alanine transaminase
AST: Aspartate transaminase
BMI: Body mass index
CHC: Chronic hepatitis C
FBG: Fasting blood glucose
FCP: Fasting C peptide
FINS: fasting insulin levels
GGT: Gamma- glutamyltransferase
HCV: Hepatitis C virus
HOMA-IR: Homeostasis model assessment of insulin resistance
HOMA-β: Homeostasis model assessment- β
ISI: Insulin sensitivity index
SVR: Sustained virological response
